# Factors contributing to decisions for giving treatment and referrals to patients with cognitive complaints in chat‐based telemedicine services in Indonesia

**DOI:** 10.1002/alz70858_100745

**Published:** 2025-12-25

**Authors:** Pukovisa Prawiroharjo, Irma Widyasari, Iskandar Purba Geraldi, Yetty Ramli, Adre Mayza, Diatri Nari Lastri

**Affiliations:** ^1^ Department of Neurology, Faculty of Medicine, Universitas Indonesia, Jakarta, Indonesia, Central Jakarta, Jakarta, Indonesia; ^2^ Dr. Cipto Mangunkusumo Public Hospital, Central Jakarta, DKI Jakarta, Indonesia

## Abstract

**Background:**

In recent years, patients in Indonesia started to actively use Chat‐Based Telemedicine Services to consult for their cognitive complaints, especially during the COVID‐19 pandemic. This study describes the characteristics of patients with cognitive complaints who used chat‐based telemedicine services in Indonesia and its correlation with the physicians’ decisions on treatment and referral.

**Method:**

This study was a retrospective cross‐sectional study during the peak of the COVID‐19 pandemic era (March 2020 to December 2021) using anonymous secondary data derived from patient chat databases on Indonesian chat‐based telemedicine services (Halodoc, Alodokter, Good Doctor, and Milvik). We applied bivariate and multivariate analysis.

**Result:**

There were 1016 patients with cognitive complaints (4/10.000 from 2.199.527 users) and most of them were females under 40 years old. Multivariate analysis showed neurologists and other specialists were more likely to give pharmacological treatment (*p* <0.01) and referral (*p* <0.01). Patients tend to get pharmacological treatment if aged ≥60 years old (*p* <0.01), were females (*p* <0.01), complained about forgetfulness (*p* <0.01), and with a history of previous diagnosis of cognitive disorders (*p* = 0.02). Patients were more likely to get a referral if there was a history of previous diagnosis of cognitive impairment (*p* <0.01), and if the patient's caregiver was the one who filed the complaints (*p* <0.01).

**Conclusion:**

In this study, we found that in a chat‐based telemedicine setting, clinicians’ decisions in providing treatment and referral were influenced by age, gender, types of cognitive complaints, history of previous diagnosis, and the complainants. Further development and research to better chat–based telemedicine services for cognitive disorder patients are needed.

